# A radiological complete response to pembrolizumab in a patient with metastatic upper urinary tract urothelial cancer and Lynch syndrome

**DOI:** 10.1002/iju5.12542

**Published:** 2022-10-28

**Authors:** Ryosuke Oki, Tetsuya Urasaki, Arisa Ueki, Kentaro Inamura, Yoshinobu Komai, Shunji Takahashi, Junji Yonese, Takeshi Yuasa

**Affiliations:** ^1^ Department of Medical Oncology, Cancer Institute Hospital Japanese Foundation for Cancer Research Tokyo Japan; ^2^ Department of Clinical Genetic Oncology, Cancer Institute Hospital Japanese Foundation for Cancer Research Tokyo Japan; ^3^ Department of Pathology, Cancer Institute Hospital Japanese Foundation for Cancer Research Tokyo Japan; ^4^ Department of Urology, Cancer Institute Hospital Japanese Foundation for Cancer Research Tokyo Japan

**Keywords:** immune checkpoint inhibitor, Lynch syndrome, metastatic urothelial cancer, mismatch repair genes, pembrolizumab

## Abstract

**Introduction:**

In Lynch syndrome, urothelial cancer is the third most common cancer, following colorectal and endometrial cancers. Little is known, however, about the efficacy of immune checkpoint inhibitors in the treatment of metastatic urothelial cancer in Lynch syndrome.

**Case presentation:**

A 49‐year‐old patient with metastatic urothelial cancer underwent pembrolizumab therapy after platinum‐containing chemotherapy. The efficacy of the pembrolizumab therapy was good. Her lung and bone metastatic lesions disappeared in imaging studies and her back pain decreased dramatically. Pathogenic mutations of *MSH2* and *BRCA2* were found in the DNA extracted from her tumor, and subsequent genetic analysis confirmed the germline pathogenic variant of *MSH2.* As such, this case was genetically diagnosed as Lynch syndrome.

**Conclusion:**

We report metastatic urothelial cancer in a patient with Lynch syndrome who demonstrated a radiological complete response to pembrolizumab therapy. Accurate genetic diagnosis can provide useful information to both the patient and their relatives.

**Abbreviations & Acronyms**
CT: computed tomography
dMMR: deficient mismatch repair
HBOC: hereditary breast and ovarian cancer
MMR: mismatch repair
MSI: microsatellite instability
mUC: metastatic urothelial cancer
UC: urothelial cancer

**Keynote message**

We report a case of metastatic urothelial cancer in a patient with Lynch syndrome that was treated with pembrolizumab after cisplatin‐based chemotherapy and demonstrated radiological complete response to pembrolizumab therapy. Genetic analyses can provide useful information for definitive diagnosis and may play a beneficial role for both the patient and their relatives.

## Introduction

Lynch syndrome is one of the most common hereditary cancer syndromes and causes various malignancies.^1^ Due to the strong association between Lynch syndrome and high levels of MSI, the treatment efficacy of the blockade of programmed death‐1 signal by immune checkpoint inhibitors is expected.^1^, ^2^

For mUC, pembrolizumab is currently the standard second‐line treatment after platinum‐based chemotherapy.^3^, ^4^, ^5^ Based on its promising anti‐tumor efficacy and manageable safety profile, the paradigm of medical treatment for patients with mUC is dramatically changing.^6^

UC is the third most common cancer in Lynch syndrome, following colorectal and endometrial cancers.^1^, ^2^ However, little is known about the efficacy of immune checkpoint inhibitors for mUC in Lynch syndrome. This is because pembrolizumab can be administered to mUC patients without investigation into the MSI status. Here, we report a case of a patient with Lynch syndrome and mUC who demonstrated a radiological complete response for pembrolizumab therapy.

## Case report

**Fig. 1:**
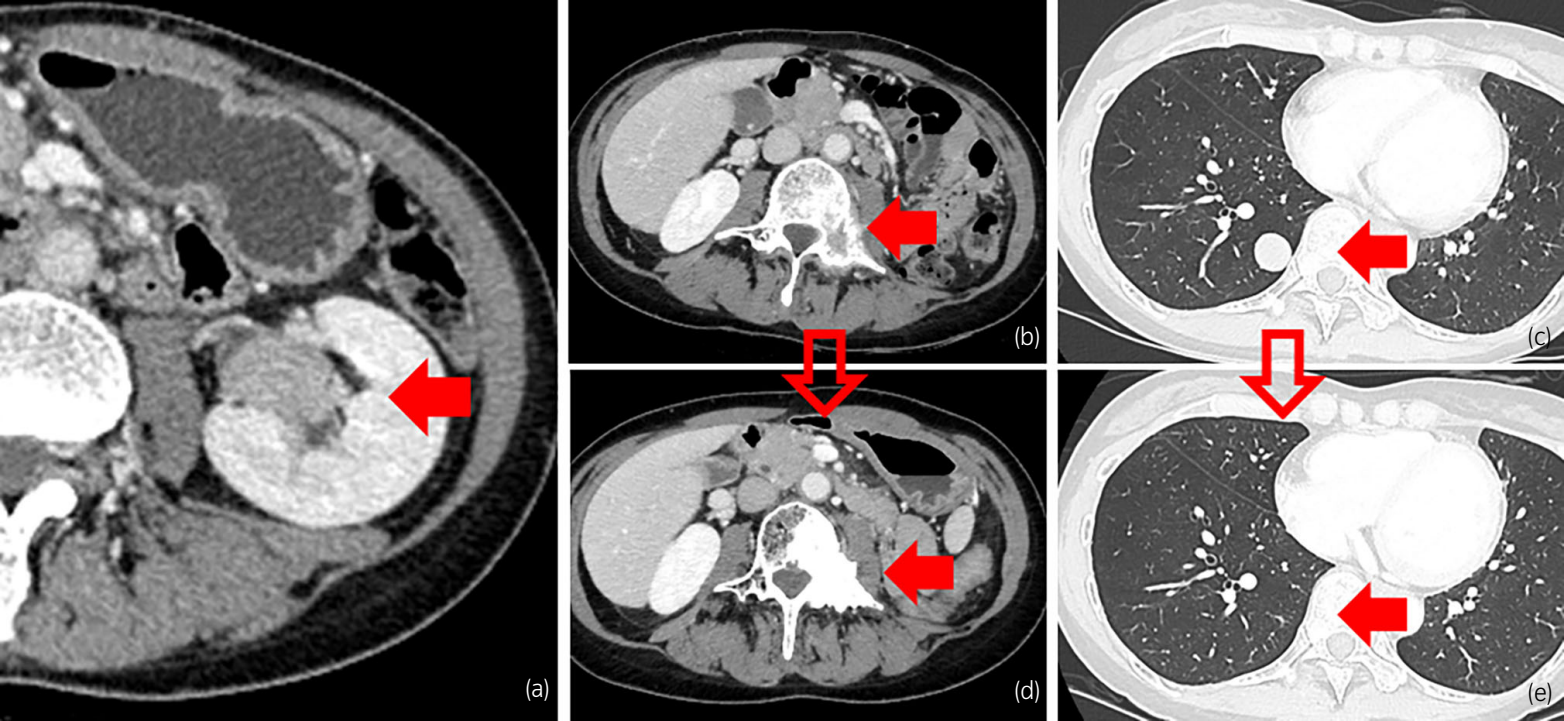
Imaging manifestation of left renal pelvic UC in Lynch syndrome. The primary left renal pelvic cancer (a) and bone and lung metastatic lesions before and after the induction of pembrolizumab therapy (bone: b, d, lung: c, e).

**Fig. 2:**
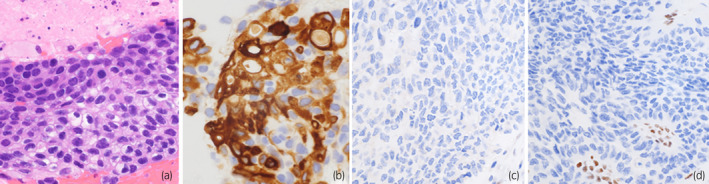
Pathological manifestation of mUC in Lynch syndrome. The pathological features of bone metastatic lesion (a: hematoxylin and eosin, ×400, b: anti‐uroplakin‐2 immunostaining, ×400) and immune‐histochemical presentation of the primary renal pelvic tumor (c: anti‐MSH2 immunostaining, ×400, d: anti‐MSH6 immunostaining, ×400).

A 49‐year‐old Japanese woman with a medical history of uterine cervical cancer and left breast cancer was diagnosed with left renal pelvic cancer (clinical stage cT2N0M0) (Fig. 1a). She underwent laparoscopic left radical nephroureterectomy. Her resected specimen was pathologically diagnosed as UC of the left renal pelvis (Grade 2, pT3, pN0, ly1, v1, INFb, RM0). Although her renal pelvic cancer was completely resected (RM0) and no nodal metastasis was found (pN0), it had invaded the lymphatics (ly1), venous vessels (v1), and peri‐pelvic fat (pT3) and was therefore considered to have a high risk of recurrence. She underwent adjuvant chemotherapy consisting of gemcitabine plus cisplatin (20% dose reduction due to her decreased creatinine clearance rate [52 ml/kg/min]). Eight months later, she noticed back pain and imaging studies revealed bone (L3 lumber vertebrae: Fig. 1b) and lung metastasis (Fig. 1c). She underwent a CT‐guided biopsy of the L3 lesion, which was pathologically and immunohistochemically (using anti‐uroplakin‐2 antibody) diagnosed as a metastasis of UC (Fig. 2a,b). She underwent palliative irradiation on the L3 metastasis to relieve her severe back pain symptoms followed by pembrolizumab immune therapy, which demonstrated a complete response after 7 cycles. The L3‐osseus and pulmonary metastases diminished on CT scan analysis (Fig. 1d,e).

In spite of this patient's relatively young age, she had three independent malignant diseases: cervical, breast, and UCs. In addition, within her third‐degree paternal relations, two gastric and two breast cancers were identified; and within her third‐degree maternal relations, one gastric, one colon, and one bladder cancer were found. Consequently, she was suspected to have a hereditary cancer syndrome. At the start of pembrolizumab administration, she underwent the cancer genome profiling test using the FoundationOne CDx (FoundationOne Medicine Inc), which demonstrated pathogenic mutation of MSH2 p.Q374* and BRCA2 p.N1784fs*7 in the DNA extraction from her tumor. Similarly, MSI‐high and TMB‐high (39 Mut/Mb) were also demonstrated. Subsequent genetic analysis disclosed the germline pathogenic variant of *MSH2*, and the *BRCA2* variant was not detected in the germline, indicating that the mutation of *BRCA2* was originated somatically. Loss of MSH2 and MSH6 expression could be also confirmed by the immune histochemical study for the primary tumor (Fig. 2c,d). On current, she continues pembrolizumab therapy with the regular following‐up protocol using thoraco‐abdominal CT scan and cystoscopy. In addition, she is undergoing regular colonoscopy surveillance for colorectal cancer. Her sister underwent cascade testing for *MSH2*, although the variant was not identified.

## Discussion

Lynch syndrome, which is one of the most common hereditary cancer syndromes, is caused by a pathogenic germline variant in one of the MMR genes, which include *MHL1*, *MSH2*, *MSH6*, and *PMS2*, or *EPCAM*.^1^, ^2^ Although Lynch syndrome is known to be associated with the most common type of hereditary colorectal cancer, the syndrome is also associated with various other malignancies including UC.^1^, ^2^, ^6^, ^7^ Based on the expected efficacy of immune checkpoint inhibitors, many clinical trials for high‐MSI/dMMR have been conducted.^1^, ^2^, ^6^, ^7^

Among these MMR genes, our case harbored the pathogenic germline variant of *MSH2*. The lifetime risk of colorectal cancer in *MSH2* pathogenic variant carriers is approximately 50%, and these patients also have a rapidly rising risk of gynecological cancers after 40 years of age in females.^8^ Periodic examination by colonoscopy has been suggested to be a useful method to detect colorectal cancer at an earlier stage and possibly reduce colorectal cancer–related mortality.^2^ In addition, immune checkpoint inhibitor therapy has certainly shed light onto the treatment of malignant diseases in Lynch syndrome.

The cancer genome profiling test in this case revealed that germline pathogenic variants were found in both *MSH2* and *BRCA2*. HBOC, which is diagnosed based on the presence of *BRCA1* or *BRCA2* pathogenic germline variants, is another one of the most common hereditary cancers.^9^ Subsequent genetic analysis revealed that the mutation of *BRCA2* was somatic. Accurate genetic diagnosis can provide useful information that enables appropriate surveillance methods for the early detection and treatment of cancers to be provided to patients and their relatives.^9^ Here, this patient was able to avoid unnecessary surveillance for HBOC.

In terms of the treatment for mUC, pembrolizumab is the currently widespread standard second‐line agent after platinum‐based chemotherapy.^3^, ^4^, ^5^ Without investigation into the MSI status, patients with mUC undergo pembrolizumab therapy in clinical practice. One systemic review reported that, among 1087 patients with upper urinary tract UC, 51 (4.7%) assumed or verified Lynch syndrome patients were identified.^10^ Results from the phase II KEYNOTE‐158 study of pembrolizumab in patients with previously treated, advanced non‐colorectal MSI‐H/dMMR cancer were reported. For the seven tumor types with greatest enrollment, which included endometrial (*n* = 49), gastric (*n* = 24), cholangiocarcinoma (*n* = 22), pancreatic (*n* = 22), small intestine (*n* = 19), ovarian (*n* = 15), and brain cancers (*n* = 13), complete responses occurred most frequently in patients with endometrial (*n* = 8) and gastric cancers (*n* = 4). This study also included five mUC patients although detailed results have not been described.^11^ In addition, although some case reports for UC treated with immune checkpoint inhibitors were found, we could not find a case with mUC in Lynch syndrome that demonstrated a radiological complete response to immune checkpoint inhibitors.^12^, ^13^

In conclusion, to the best of our knowledge, this is the first report of an mUC patient with bone and lung metastases in Lynch syndrome, who demonstrated a radiological complete response to immune checkpoint inhibitor therapy. Genetic analyses can provide useful information for definitive diagnosis and can also play a beneficial role for both the patient and their relatives.

## Author contributions

Oki Ryosuke: Conceptualization; data curation; visualization; writing – original draft. Tetsuya Urasaki: Conceptualization; data curation. Arisa Ueki: Supervision; writing – review and editing. Kentaro Inamura: Supervision; visualization; writing – review and editing. Yoshinobu Komai: Data curation. Shunji Takahashi: Supervision. Junji Yonese: Supervision. Takeshi Yuasa: Conceptualization; data curation; supervision; visualization; writing – review and editing.

## Conflict of interest

The authors declare no conflict of interest.

## Approval of the research protocol by an Institutional Reviewer Board

Not applicable.

## Informed consent

Written informed consent was obtained from the patient for publication of this case report.

## Registry and the Registration No. of the study/trial

Not applicable.
